# Performance Prediction of Differential Fibers with a Bi-Directional Optimization Approach

**DOI:** 10.3390/ma6125967

**Published:** 2013-12-18

**Authors:** Yi Wang, Yongsheng Ding, Kuangrong Hao, Tong Wang, Xiaoyan Liu

**Affiliations:** 1 College of Information Sciences and Technology, Donghua University, Shanghai 201620, China; E-Mails: violet0826@163.com (Y.W.); krhao@dhu.edu.cn (K.H.); xiaotong@mail.dhu.edu.cn (T.W.); liuxy@dhu.edu.cn (X.L.); 2 Engineering Research Center of Digitized Textile & Fashion Technology, Ministry of Education, Donghua University, Shanghai 201620, China

**Keywords:** bi-directional prediction, neural networks, multi-objective evolutionary algorithm, performance prediction, differential fibers

## Abstract

This paper develops a bi-directional prediction approach to predict the production parameters and performance of differential fibers based on neural networks and a multi-objective evolutionary algorithm. The proposed method does not require accurate description and calculation for the multiple processes, different modes and complex conditions of fiber production. The bi-directional prediction approach includes the forward prediction and backward reasoning. Particle swam optimization algorithms with K-means algorithm are used to minimize the prediction error of the forward prediction results. Based on the forward prediction, backward reasoning uses the multi-objective evolutionary algorithm to find the reasoning results. Experiments with polyester filament parameters of differential production conditions indicate that the proposed approach obtains good prediction results. The results can be used to optimize fiber production and to design differential fibers. This study also has important value and widespread application prospects regarding the spinning of differential fiber optimization.

## 1. Introduction

It is well known that before the fiber production line starts, the production parameters must be determined. If the fiber performance needed is changed, the corresponding production parameters must be changed, too. If there is an approach to find the relationship between fiber performance and production parameters, the fiber production can be optimized. However, the fiber production line is a large-scale production system that has multiple processes, different modes and complex conditions, so it is difficult to complete the above task. Since the 1960s, a large amount of basic theory research has been applied on fiber production. The traditional optimization methods of this system involve controlling the production equipment, improving the production processes and optimizing the fiber performance. However, most of them are applied to controlling production, such as the control of winding machines [1], the coagulation bath [2] and the stretching process [3]. In recent years, there has been some research in building mathematic models of the production process, which uses simulation technologies to find the accurate description and calculation for every step or a part of the fiber production, but not the whole process. Tan tried to find the relationship between diameter distributions and the viscosity and elasticity of meltblown fibers [4]. Gou gave a two-dimensional model of dry spinning polymer fibers [5]. Lee gave a numerical reduction model of optical fibers [6]. Kadi gave a review of the influence from mechanical behavior [7]. Arafeh used a neuro-fuzzy logic approach to model the material process [8]. Although these research results are accurate and correct, they have not considered the interaction influence among multiple steps. In addition, if the production mode or the equipment condition is changed, this research must be re-modified in order to adapt to the new production process.

In order to find the relationship between the fiber performance and production parameters in the method of a black box, neural networks and multi-objective evolutionary algorithm are mentioned here, and both algorithms have been applied on industrial problems successfully for many years. There are some reviews for different neural networks [9,10,11,12,13]. The multi-objective evolutionary algorithm is an area for multiple criteria decision making, which is used to make an optimal decision in the presence of trade-offs between two or more conflicting objectives. It has been successfully used in pattern recognition, adaptive control and prediction problems [14,15]. Some similar research in this field has been done, such as Liu adopting an adaptive neuro fuzzy inference system (ANFIS) to perform parameter prediction [16], Deng using intelligent decision support tools to design production [17], Yu using a fuzzy neural network to predict the fabric hand [18] and Yang using a neural network approach to optimize the mechanical characteristics of short glass fiber [19]. However, most of the existing results about prediction problems are always a matter of one-way prediction. 

Based on neural networks and a multi-objective evolutionary algorithm, this paper develops an approach to predict both fiber performance and production parameters, and its final target is to optimize fiber production. This approach only depends on the production parameters and their corresponding fiber performance, so it remains unaffected when the production modes or the equipment conditions are changed. The prediction in this paper consists of the bi-directional prediction process, which includes the prediction of the fiber performance by the production parameters and the prediction of the production parameters by the fiber performance. If these two prediction processes are regarded as two irrelevant parts, they can be solved by many kinds of algorithms, but there may be some problems. Because the forward prediction is based on the production process, it is a forward process in nature, which means it can be seen as an independent prediction. However, backward reasoning is complex, because it is a reverse process in nature. For example, different production parameters can achieve same fiber performance, and this situation will affect the backward reasoning results, which means that one-way prediction is not enough to solve this backward reasoning. In a word, comparing with the forward prediction, which can be solved by different algorithms simply, backward reasoning is too complex to be realized by the same algorithms as the forward prediction, because of the missing data, the conflict data, the interaction influences and other problems. In order to avoid those problems, the forward prediction and backward reasoning are designed together in the bi-directional prediction approach in this paper, and in addition, the prediction approach adopted in this paper is a bi-directional prediction approach, whose backward reasoning is based on the forward prediction.

In order to solve the accuracy problem, through experiments and data analysis, this paper uses hybrid intelligent algorithms, including a particle swarm optimization algorithm with a K-means algorithm to optimize the clustering and to increase the accuracy of the forward prediction. Backward reasoning, as mentioned before, is based on the forward prediction, and it can be seen as a multi-objective evolutionary problem. This paper uses a multi-objective evolutionary algorithm, the clustering results and the forward prediction to achieve the backward reasoning results.

The remainder of this paper is organized as follows. In Section 2, we give the introduction of the fiber production and the bi-directional prediction optimization, including the production process, the production parameters, the fiber performance and the design of the forward prediction and backward reasoning. In Section 3, the implementation, simulation results and error analysis of the bi-directional prediction approach are given, which is applied to design polyester filament parameters. Finally, concluding remarks are given in Section 4.

## 2. Fiber Production and Bi-Directional Prediction Optimization

### 2.1. Fiber Production Process 

**Figure 1:**
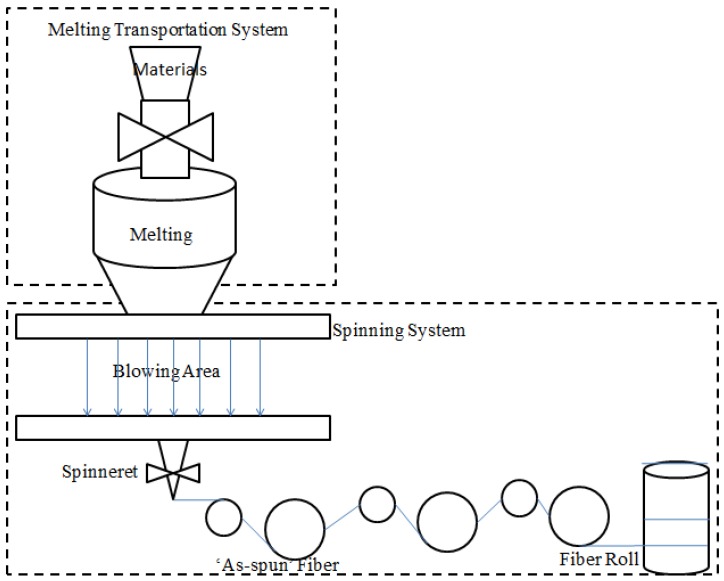
The process of fiber production.

The production of fiber is a complex production line, which generally consists of two systems: the melting transportation system and the spinning system. The melting transportation system is used to convert the fiber materials to liquid, and this is accomplished by the following spinning system. The spinning system includes a quenching area and a stretching process. The quenching area is used to help the liquid to solidify in the streams, which are called ‘as-spun’ fibers [20,21], and then, the ‘as-spun’ fibers can be stretched in the stretching process, according to the stretching ratio. Although different fibers ask for different combinations of equipment and materials, the basic production processes are similar. The production process of polyester staple fiber is depicted in Figure 1. 

In order to produce fibers with good performance, the optimization of fiber production always depends on the adjusting and controlling of production parameters. Because the production line consists of several processes, there are numerous production parameters that need to be determined in every process. In addition, all the processes have interaction influences on each other. The traditional approaches, which follow experience, are not accurate enough to solve the problems above, and the mathematic models are not adaptable enough to solve for differential fibers. Therefore, if we can find a way to achieve the bi-directional prediction results with hybrid intelligent algorithms, the production parameters can be determined and fiber production can be optimized. 

First of all, the selection of the key production parameters and fiber performance is the foundation of this paper. At the beginning of production, the materials will be melted into liquid according to a predefined viscosity and temperature; this process belongs to the chemical category, so it is not taken into account in this paper. After this process, the materials will enter the quenching area and the stretching process. These two processes are the most important ones in this paper, so all the parameters of these processes are considered in the bi-directional prediction approach.

In the bi-directional prediction approach, the prediction of fiber performance by production parameters is a forward prediction process and the prediction of production parameters by fiber performance is a backward reasoning process.

### 2.2. Fiber Production Process Overall Design of Bi-Directional Prediction Approach

As mentioned above, the bi-directional prediction approach consists of the forward prediction and backward reasoning.

#### 2.2.1. Forward Prediction

Because fiber production is a positive process, the forward prediction is a positive process, too. It stands to reason that when the parameters of fiber production are changed, the corresponding fiber performance will be changed accordingly. In addition, production is a long-term process, which means that the predetermined production parameters usually need not be changed once in production. Because of the production conditions and environment conditions, there must be a small disturbance between the real production parameters and the presupposed production parameters. In other words, the real measured production parameters of production, at the same time, are not the same, but they are similar. Therefore, if the production parameters are similar, there must be little difference between the fiber performances. Meanwhile, though the production parameters of every production process are unstable, the fiber performance must also have incredibly small variations, which means they all have the characteristic of aggregation.

From the above analysis, the relationship between the production parameters and the fiber performance can be assumed as a many-to-many problem. Because there is a characteristic of aggregation in the production parameters and their corresponding fiber performance, this many-to-many problem can be simplified to a one-to-one problem by a clustering algorithm. In order to solve this one-to-one problem, this paper developed neural networks based on a clustering algorithm to fulfill the function of the forward prediction.

#### 2.2.2. Backward Reasoning

The prediction of production parameters by fiber performance is a reverse process, so the prediction process can be seen as backward reasoning. Compared with the forward prediction process, the backward reasoning process is more complex. Traditional prediction approaches will meet some problems, as follows. Firstly, the variety of fiber performance is more inadequate than the production parameters. Secondly, because the production has several processes and every process of the whole production is not independent of each other, it is possible that similar fiber performance can sometimes be achieved with different production parameters, which means that backward reasoning cannot be simplified to a one-to-one problem; it is a one-to-many problem. The one stands for the fiber performance, and the many stands for the production parameters. Therefore, traditional neural networks based on a clustering algorithm, which is used in the forward prediction process, are not suitable for backward reasoning.

In order to solve the above problems, backward reasoning consists of not only itself, but also the clustering results and the forward prediction. First, we use the clustering results as the foundation of backward reasoning. Since the clustering results are based on the production parameters, it can keep the variety of fiber performance without changing its structure and avoid the influences of the missing and conflict data. Secondly, in order to solve the one-to-many problem, the multi-objective evolutionary algorithm is used in it to find the optimal answer in the many part, and the forward prediction is used to calculate the objectives. 

**Figure 2:**
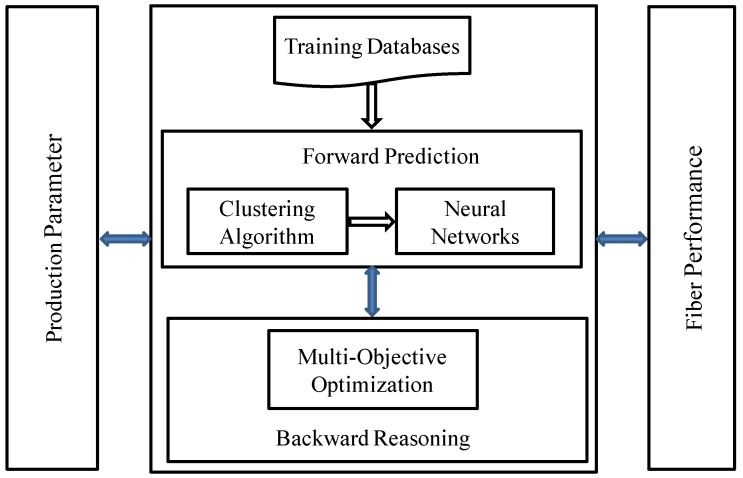
The overall chart of the bi-directional prediction approach.

Figure 2 is the overall chart of the bi-directional prediction approach above.

### 2.3. Design and Improvement of the Bi-Directional Prediction Approach

The design and improvement of the bi-directional prediction approach will be divided into three parts: the clustering process, the forward prediction and backward reasoning.

#### 2.3.1. Design of the Clustering Process

As mentioned above, the bi-directional prediction is composed of the forward prediction and backward reasoning. Backward reasoning depends on the forward prediction, and the forward prediction is based on the positive clustering result; so, the clustering result is the most important point in the bi-directional prediction approach.

The clustering algorithm is an important tool for data analysis and an unsupervised classification algorithm, which can classify the unlabeled data automatically. Up to now, a lot of algorithms have been proposed and applied in clustering problems, but they all have a common problem, that the result is the locally optimal solution. In order to solve this problem, this paper uses some hybrid intelligent algorithms to optimize traditional clustering algorithms. Compared with other clustering algorithms, the particle swam optimization algorithm (PSO) with its multifarious particles can get the global optimal solution more efficiently [22,23], and the K-means algorithm is good at optimizing the local optimal solution [24,25]. 

Firstly, the whole clustering process will be operated by every kind of fiber performance, which can optimize the clustering results. All the training data are preprocessed to be the same order as Equation (1); this normalization approach can avoid the effects of the difference across different data kinds and increases the clustering efficiency and accuracy. The preprocess effect is relieved by Equation (2), where X^*^ is the initial data and *X* is the processed data.

(1) $$X=\frac{X-\text{min}\left(X^{*}\right)}{\text{max}\left(X^{*}\right)-\text{min}\left(X^{*}\right)}$$

(2) $$X=X\times \left(\text{max}\left(X^{*}\right)-\text{min}\left(X^{*}\right)\right)+\text{min}\left(X^{*}\right)$$

Secondly, we use the PSO function and multifarious particles to find the preliminary result. The selection of the initial particles of the PSO function is random in the training data, and the initial particles include the input data and the output data of every center. The selection in training data can avoid the generation of invalid centers, and including the output data can avoid the error of data mutations. The fitness of the PSO function is the key point, and the calculation of the *i-*th fitness is as shown in Equation (3), where *x_j_* is the *j-*th training input data, *n* is the number of training input data, *X_i_* is the *i-*th particle and *m* is the number of particles. $\left\|x_{j}-X_{i}\right\|$ is the calculation of the Euclidean distance between *x_j_* and *X_i_*.

(3) $$fitness(i)=\frac{\sum_{j=1}^{n}\text{min}{\;\|x_{j}-X_{i}\|}^{2}}{n},i=1,2...m$$

Thirdly, the particle updating functions are as shown in Equation (4), where *w* is the speed factor, *r*_1_ and *r*_2_ are random numbers between 0 and 1, *P*_best_ and *G*_best_ are the best particle and the global best particle, respectively, *t* is the step number of this updating and *n*_step_ is the total iterative times of the whole clustering process. Equation (4) shows the traditional particle updating functions [22].

(4) $$\begin{array}{l} V_{k+1}=wV_{k}+c_{1}r_{1}(P_{\text{best}}-X_{k})+c_{2}r_{2}(G_{\text{best}}-X_{k})\\ w=w_{\text{max}}-(w_{\text{max}}-w_{\text{min}})t/n_{\text{step}}\\ X_{k+1}=X_{k}+V_{k+1} \end{array}$$

Through iterative analysis and calculation, all the particles will be directed to the best result, which means that most of the particles will be similar to each other. When the similarity of the particles reaches a certain high degree, the best particle is nearer to the global optimal solution. However, all the initial particles are different in their centers’ order with respect to each other, so the similarity is difficult to judge by the particles themselves. However, the fitness of every particle stands for both the cluster accuracy and its particle, so the similarity of the fitness can stand for the similarity of the particles. Then, the *K*-means function is added to increase the accuracy of the optimal solutions and to optimize the clustering result.

In addition, a mutation operator is used to make particles that are far from the local optimal solution. Because the mutation operator can lead the particles to be both better and worse, the mutation operator is used on all the particles, except the optimal particle. Then, the optimal particle can just keep being optimized.

Finally, we calculate the output centers according to the input centers and the training output data, as shown in Equation (5), where *Y_j_* is the *j-*th output center, *X_j_* is the *j*-th input center, *x_i_* is the training input data, which are clustered into the *j-*th input center, *y_i_* is the corresponding training output data of *x_i_* and *n* is the number of training data in the *j-*th center. The numerator of the coefficient calculation is the exponent of the distance between the input data and the input center, and its denominator is the total of all the distance factors. This cannot only keep the inverse proportion between the coefficient and the distance, but also avoid the effect from the coefficients’ order of magnitude. In this function, the closer the input data is to the input center, the bigger the coefficient of the corresponding output data will be weighted, which means it can decrease the effect from some data that have similar production parameters and performance with a large difference.

(5) $$Y_{j}=\frac{\sum_{i=1}^{n}y_{i}\text{exp}\left[-\frac{{\left\|x_{i}-X_{j}\right\|}^{2}}{2}\right]}{\sum_{i=1}^{n}\text{exp}\left[-\frac{{\left\|x_{i}-X_{j}\right\|}^{2}}{2}\right]}$$

The steps of the clustering algorithm are as follows.

Step 1: Preprocess the training data by Equation (1).
Step 2: Select the initial particles randomly in the training data and calculate the initial fitness by Equation (3) of all the particles and the initial *P*_best_ and *G*_best_ with their particle as the local and global optimal one.
Step 3: Update the particles by Equation (4) and make sure that all velocities of particles are less than *V*_max_.
Step 4: Calculate the fitness of all the particles by Equation (3) and update *P*_best_ and its local optimal particle. If *P*_best_ < *G*_best_, then *G*_best_ = *P*_best_, and update its globally optimal particle.
Step 5: Regard the *P*_best_ particle as the optimal particle; other particles have the mutation operator. The rate of the mutation operator is 10%, and the mutations will change into another particle selected in the training data randomly.
Step 6: If all the steps are over, or two thirds of the fitness values are similar to the global best particle’s, then use the K-means function to optimize the global best particle three times; else, return to Step 3.
Step 7: Calculate the output centers as in Equation (5) by the global best particle, and output the clustering result. During the calculation, if there is no training data in one center, then this center will be deleted from the final clustering result.

#### 2.3.2. Design and Improvement of the Neural Networks

Traditional neural networks can do prediction, but this prediction is point prediction, which conveys little information about the prediction accuracy [10,11]. Radial basis function neural networks based on a Gaussian function are used here to optimize the prediction accuracy and avoid the point prediction [26,27]. There are some traditional difficulties in this kind of algorithm: the calculation of σ and the calculation of weight [7,9]. 

Firstly, the value of σ in the radial basis function neural networks shows the width of each center, and the final fitness value of each clustering result shows the average distance of all the centers. Therefore, this paper uses the final fitness value of each clustering result to calculate the value of σ by Equation (6), where *m* is the number of kinds of fiber performance, and factor 2 enlarges the width of every center by the average distances as the maximum distance.

(6) σ(i)=2×fitness(i),i=1,2…m

Secondly, the traditional function to calculate the weight of every output center relies on the value of σ. Although the calculation of σ is improved and simplified, in this paper, it is an average value. In order to reduce the prediction errors, this paper also improves the function of weight value, which can assure that the total value of all the weights is one and the prediction value is within the range. The traditional Gaussian function is as shown in Equation (7), while the improved Gaussian function is as shown in Equation (8), where *w_ij_* is the *j-*th output center’s weight of the *i-*th output kind, *X* is the input data, *C_ij_* is the *j-*th input center of the *i-*th output kind, *σ* is a constant, *m* is the number of output kinds and *n* is the clustering amount.

(7) $$w_{ij}=\text{exp}\left[-\frac{{\left(X-C_{ij}\right)}^{2}}{2\sigma^{2}}\right],i=1,2...m,j=1,2...n$$

(8) $$w_{ij}=\frac{\text{exp}\left[-\frac{{\left(X-C_{ij}\right)}^{2}}{2\sigma^{2}}\right]}{\sum_{j=1}^{N}\left[-\frac{{\left(X-C_{ij}\right)}^{2}}{2\sigma^{2}}\right]},i=1,2...m,j=1,2...n$$

The steps of the clustering algorithm are as follows.

Step 1: Preprocess the input production parameters by Equation (1).
Step 2: Calculate the σ by Equation (5) and the weights by Equation (7).
Step 3: Add up the production of every center and its weight.
Step 4: Relieve the preprocess effect by Equation (2), and get the prediction result of the fiber performance.

#### 2.3.3. Design and Improvement of the Multi-Objective Evolutionary Algorithm

Backward reasoning is a one-to-many problem; in other words, there is more than one production parameter set that can achieve the target fiber performance. As mentioned above, the foundation of backward reasoning is the positive clustering result based on output classes. Therefore, every clustering result can be seen as a subset, and every local optimal solution can be seen as a sub-goal. A multi-objective evolutionary algorithm is a good tool to solve this kind of problem and find the globally optimal solution [28,29]. The non dominated sorting genetic algorithm (NSGA)-II [28,30] algorithm has been one of the most popular algorithms to solve this kind of problems in recent years.

The backward reasoning process based on the multi-objective evolutionary algorithm is shown as follows:

Step 1: According to the input data in backward reasoning, select the high similarity output centers and their corresponding input centers in the positive clustering results by the classes of the output data and save the deviations between the input data and the centers. All of the corresponding input centers are candidate solutions.
Step 2: Optimize all of the candidate solutions by the selection process, the genetic operator and replacement process. Calculate the fitness of every objection by Equation (9) of the solution, where *S_ij_* is the *j-*th output of the *i-*th solution’s forward prediction result, *X_j_* is the *j-*th output of the input fiber performance, *m* is the number of the solutions and *n* is the number of fiber performance kinds.
  (9) $$fitness(j)=abs(S_{ij}-X_{j}),i=1,2\ldots m,j=1,2\ldots n$$
Step 3: Select the better solutions to be the optimized solution sets by two rules. Firstly, calculate the better number of other solutions, which is in total better than this solution, solution by solution. The in total better solution means that every fitness value in it is smaller than the corresponding fitness value in this solution. Secondly, from the better numbers, small to big, calculate the total value of all the fitness solutions by the solutions that have the same better number, and choose the one that has a smaller value to fulfill the new solution set, until the set is full.
Step 4: If all the steps are over, then relieve the preprocess effect and output the final solution set; else, return to step 2.

**Figure 3:**
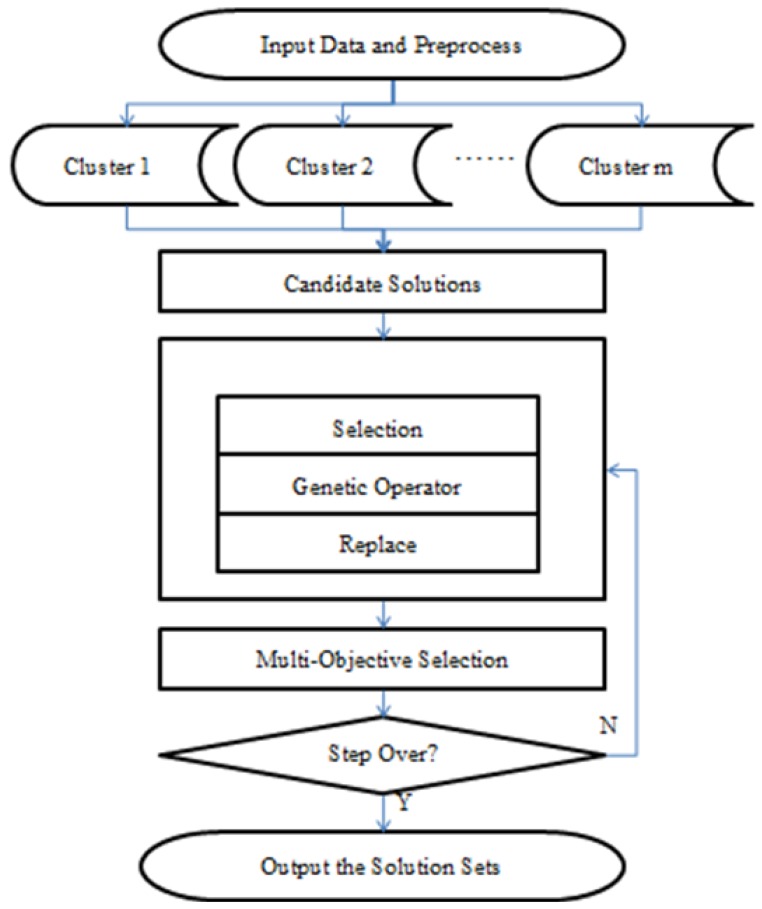
The overall chart of backward reasoning.

Figure 3 is the overall chart of the algorithm above.

## 3. Application and Results 

The bi-directional prediction approach consists of three parts, the modified clustering algorithms, the forward prediction and backward reasoning. Therefore, the application of this prediction approach will be tested in those three parts.

### 3.1. The Performance of the Clustering Approach

As mentioned above, the forward prediction is based on the clustering results, and backward reasoning is based on the forward prediction; the modified clustering algorithm is the foundation of the bi-directional prediction approach. The accuracy and veracity of this modified clustering algorithm is an important problem. Because the accuracy and veracity of fiber production parameters have no judgment standard, the iris data is used to test this algorithm. Iris data, which has three classes, 150 data rows and four columns, is always used in the performance test of a clustering algorithm.

Table 1 lists the parameters of the algorithm applied in this paper, and Table 2 is the error list and the fitness of the proposed algorithm, the traditional PSO algorithm and the K-means algorithm. According to the results, the proposed algorithm has accuracy and veracity of high levels, and the clustering result by this algorithm can also optimize the prediction accuracy.

**Table 1:** Parameters of the algorithm applied.

Category	Item	Value
Parameters Value	Learning Factor (*C*_1_,*C*_2_)	1.5
	Speed Factor (*w*_max_)	0.9
	Speed Factor (*w*_min_)	0.4
	Velocity Maximum	0.5
	Maximum Step	100
	Classify Number	3
	Particle Number	10
Data Size	Size of Training Input Data	(150 × 4)
	Size of Training Output Data	(150 × 1)
	Size of Input Centers	(3 × 4 × 1)
	Size of Output Centers	(3 × 1)

**Table 2:** Clustering results of different algorithms. PSO, particle swam optimization algorithm.

Item	PSO	K-means	This Paper
Error	15	17	6
Final Fitness	0.67	0.53	0.27

### 3.2. The Prediction Results of Polyester Staple Fiber

#### 3.2.1. Parameters Selection 

As mentioned before, the most important sections of fiber production are the quenching area and the spinning system, and the material melting is assumed to be predetermined in this paper. The experiments are based on a 1.56 dtex (its fineness is 1.56 dtex) cotton-type polyester staple fiber with a fully-closed quenching area fiber production line. Since there are many production lines, with different production parameters, there are a large number of production parameters and their corresponding fiber performances. Table 3 is a parameters list with the value ranges of these fiber production lines. In Table 3, the major variable production parameters are the spinning velocity (SV), the spinning temperature (ST), the quenching velocity (QV) and the quenching temperature (QT). In addition, the major variables of fiber performance were the elongation, corresponding to 1.5 times the yielding stress (EYS1.5), the coefficient of variance (EYSCV), the breaking tenacity (BT) and the ability for elongation when broken (BE).

**Table 3:** Clustering results of different algorithms. EYSCV, elongation yielding stress coefficient of variance; BT, breaking tenacity; BE, ability for elongation.

Category	Item	Value
Fiber Category	Fineness (dtex)	1.56
	Post-drawing Ratio	3.6523
Equipment Parameters	Non-quenching Gap Height (cm)	6
	Number of Spinneret Orifice	3064
	Diameter of Spinneret Orifice (cm)	0.0022
	Pump Mass Throughput (g/min·hole)	0.0097
Spinning Parameters	Spinning Velocity (m/min)	1000~1197
	Spinning Temperature (°C)	280~299
	Characteristic Viscosity (dL/g)	0.63
Quenching Parameters	Quenching Velocity (m/min)	100~139
	Quenching Temperature (°C)	20~24
Fiber Performance	EYS1.5	196.29~237.78
	EYSCV	5.46~10.04
	BT	5.82~6.81
	BE	20.94~24.05

#### 3.2.2. Design of the Bi-Directional Prediction Approach 

All of the practical fiber data are divided into two parts. About 3000 of them are selected as training data, and the others are the testing data. Table 4 lists the parameter setting of different parts of the bi-directional prediction approach.

**Table 4:** Parameters of the bi-directional prediction approach.

Category	Item	Value
Forward Prediction	Learning Factor (*C*_1_,*C*_2_)	1.5
	Speed Factor (*w*_max_)	0.9
	Speed Factor (*w*_min_)	0.4
	Velocity Maximum	0.5
	Maximum Step	100
	Classify Number	350
	Particle Number	10
	Size of Training Input Data	(3000 × 4)
	Size of Training Output Data	(3000 × 4)
	Size of Input Centers	(350 × 4 × 4)
	Size of Output Centers	(350 × 4)
	Size of Weights	(350 × 4)
Backward Reasoning	Population Size	40
	Generations Number	1000
	Objectives Number	5
	Variables Number	4

#### 3.2.3. Results and Analysis

(1) Clustering Results

The clustering results are the foundation of forward prediction and backward reasoning. Its accuracy and rationality have direct influences on the bi-directional prediction results. In order to prove that the clustering results are accurate and reasonable, other algorithms are used to compare with it. The clustering results, including the final classification number and the fitness, are as shown in Table 5.

**Table 5:** Clustering results of different algorithms.

Item	PSO	K-means	This Paper			
			EYS1.5	EYSCV	DT	DE
Centers	213	160	343	345	344	344
Fitness	0.140	0.087	0.049	0.048	0.045	0.049

All the original classification numbers are set to 350. Through the clustering process, some centers will be deleted, because they are too alike other centers. In the proposed algorithm, the clustering process is repeated four times, because the corresponding output data has four kinds. The final center number of the proposed algorithm is twice as much as that of the traditional algorithms. It can be said that the proposed algorithm keeps the variety of the centers. Table 6 lists the comparison of clustering results with different algorithms; some data are classified in the same center by traditional algorithms, while by the proposed algorithm, they are classified in different centers, where all the data are preprocessed by Equation (1). 

**Table 6:** The center number of different algorithms. SV, spinning velocity; ST, spinning temperature; QV, quenching velocity; QT, quenching temperature.

Training Data					Center Nubmer		
SV	ST	QV	QT	DE	PSO	K-means	This paper
0.12	0.06	0.99	0.46	0.62	6	23	153
0.01	0.27	0.99	0.59	0.74	6	23	169
0.04	0.22	0.74	0.46	0.68	6	23	234
0.09	0.11	0.99	0.46	0.66	6	23	305

In addition, the fitness of all algorithms is the total value of the difference of the training data and its corresponding center, so the smaller the fitness is, the better the clustering result is. It can be said that the proposed algorithm in this paper has a good clustering result.

(2) Forward Prediction Results

The fiber performance, including EYS1.5, EYSCV, DT and DE, can be predicted by forward prediction with the production parameters consisting of SV, ST, QV and QT.

**Figure 4:**
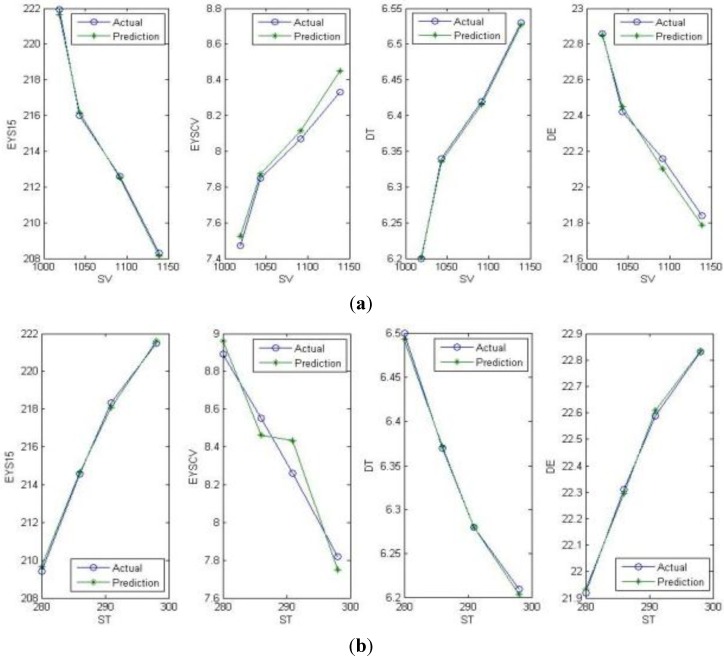

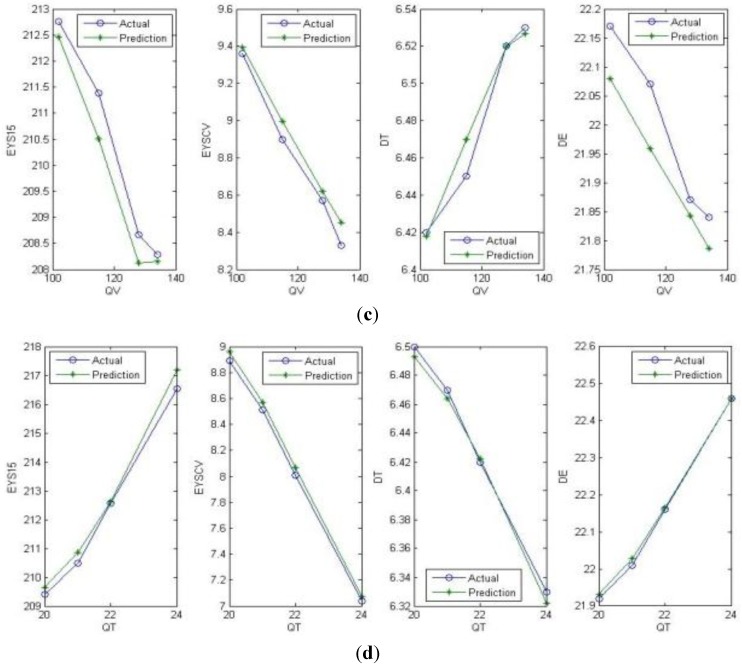
(**a**) Prediction results relying on SV; (**b**) prediction results relying on ST; (**c**) prediction results relying on QV; (**d**) prediction results relying on QT. In order to prove that the forward prediction algorithm has a good result generally and that it can be adapted to differential fibers, we use more datasets from the experiment above and another experiment’s data to test it. The training data is about 3,000 datasets, and the testing data is about 300 datasets. Table 8 is the average error of both fibers. The errors of the differential fibers can be acceptable, and these results can lay a good foundation for backward reasoning.

The production parameters as the input data are listed in Table 7, in which one parameter is regarded as the variable and the others are the invariant parameters. Figure 4 shows the actual values and the prediction results of different parameters, respectively. Some prediction results in Figure 4 may have a bigger error than others, but this is mostly because of the y-range; so, the forward prediction achieves good results.

**Table 7:** Prediction parameters of the input.

Number	SV	ST	QV	QT
(a)	Variable	290	134	21
(b)	1050	Variable	130	20
(c)	1140	290	Variable	21
(d)	1050	280	130	Variable

**Table 8:** Forward prediction results of differential fibers.

Error (%)	EYS15	EYSCV	DT	DE
Fully-closed	0.28	0.74	0.21	0.20
Semi-open	0.26	0.64	0.22	0.20

(3) Backward Reasoning Results

The production parameters, including SV, ST, QV and QT, can be predicted by backward reasoning with the fiber performance consisting of EYS15, EYSCV, DT and DE.

From Figure 4, it can be observed that there is a variation tendency in every relationship between production parameters and fiber performance. For example, the increase of ST and QT will increase EYS15 and DE, while reducing EYSCV and DT. The increase of SV and ST will increase DE, while reducing EYS15 and DE. EYSCV will be increased by SV and reduced by QV. This also shows that backward reasoning is a one-to-many problem.

In this paper, there are four kinds of fiber performance, and there is no special order for each performance. Therefore, four objective function values will be averaged. Table 9 lists several backward reasoning results of the 1.56 dtex cotton-type polyester staple fiber with a fully-closed quenching area fiber production line. All of the experiment data is based on practical fiber production. Most of the backward reasoning errors are below 5%, and the average error is around 2%. As mentioned before, fibers of a similar performance can have different production parameters. Table 10 lists the backward reasoning of similar performance. The top part of Table 10 lists some examples of similar performance and their corresponding parameters. There are differences of output among N1, N2 and N3, which have similar input. After giving the input of N1, because backward reasoning has not only converging speed, but also diversity, it will have several solutions of one problem when that is necessary. Finally, the solutions of backward reasoning include S1, S2 and S3. S1 is the best solution of this prediction; however, S2 and S3 are also feasible answers, because following N2 and N3, these two production parameters can achieve similar fiber performance. Table 10 shows that the proposed algorithm does well in this backward reasoning.

**Table 9:** Prediction results of backward reasoning.

Number	**Input**				**Actual**				**Predict**			
	**EYS1.5**	**EYSCV**	**DT**	**DE**	**SV**	**ST**	**QV**	**QT**	**SV**	**ST**	**QV**	**QT**
1	217.31	7	6	23	1074	290	22	134	1069.8	289.6	22.0	136.5
2	205.28	8.94	6.6	21.61	1182	286	22	117	1196.7	285.8	22.0	117.2
3	217.84	8.6	6.3	22.56	1095	297	22	139	1094.2	296.8	22.0	138.7
4	214.63	8.25	6.37	22.32	1090	282	23	113	1077.3	280.0	23.0	113.7
5	214	7.23	6.39	22.27	1057	282	23	139	1078.0	283.5	23.5	138.6
6	223.62	6.49	6.16	22.99	1103	296	24	131	1054.7	288.7	23.7	137.0
7	202.2	10.04	6.67	21.38	1190	287	20	110	1190.4	281.5	20.0	106.3
8	205.93	9.87	6.58	21.66	1197	292	20	107	1165.9	284.3	20.2	106.3
9	225.81	8.38	6.11	23.15	1041	297	20	112	1030.5	290.9	21.1	108.8
10	223.90	7.81	6.15	23.01	1087	292	23	107	1089.1	292.3	22.9	108.3
Error (%)									1.65	1.11	1.34	1.85

**Table 10:** Prediction results of backward reasoning.

Item	**Number**	**Input**				**Output**			
		**EYS15**	**EYSCV**	**DT**	**DE**	**SV**	**ST**	**QV**	**QT**
Real Data	N1	223.90	7.81	6.15	23.01	1087.00	292.00	23.00	107.00
	N2	224.92	7.79	6.13	23.09	1079.00	292.00	23.00	106.00
	N3	225.47	7.86	6.11	23.13	1047	292	22	111
Solution	S1	223.90	7.81	6.15	23.01	1089.08	292.33	22.87	108.34
	S2					1079.08	291.33	22.78	105.34
	S3					1045.10	289.99	22.04	111.93

## 4. Conclusions 

A newly bi-directional prediction approach based on hybrid intelligent algorithms, which involved neural networks and multi-objective evolutionary algorithm, is adopted and further developed in this paper. The bi-directional prediction approach makes an attempt to find the relationship between the production parameters and the fiber performance of differential fiber production lines and optimizes the production of the fiber. In addition, the parameters of the bi-directional prediction approach can be changed to suit differential fibers. The predictions of differential fibers given in this paper prove the accuracy of this approach. This approach can provide a way to determine the parameters before real production and to meet different types of fiber performance.
